# Phenotypic and genetic dissection of component traits for early vigour in rice using plant growth modelling, sugar content analyses and association mapping

**DOI:** 10.1093/jxb/erv258

**Published:** 2015-05-28

**Authors:** M. C. Rebolledo, M. Dingkuhn, B. Courtois, Y. Gibon, A. Clément-Vidal, D. F. Cruz, J. Duitama, M. Lorieux, D. Luquet

**Affiliations:** ^1^CIAT, Agrobiodiversity, AA 6713, Cali, Colombia; ^2^IRRI, CESD Division, DAPO Box 7777, Metro Manila, Philippines; ^3^CIRAD, UMR AGAP, F-34398 Montpellier, France; ^4^IRD, DIADE Research Unit, Institut de Recherche pour le Développement, 34394 Montpellier Cedex 5, France; ^5^INRA, Metabolome Platform of UMR 1332, Bordeaux, France

**Keywords:** Genome wide association study (GWAS), genotyping by sequencing, heuristic approach, model-assisted phenotyping, *Oryza sativa* L., sugar metabolism.

## Abstract

Early vigour of rice, defined as seedling capacity to accumulate shoot dry weight (SDW) rapidly, is a complex trait. It depends on a genotype propensity to assimilate, store, and/or use non-structural carbohydrates (NSC) for producing large and/or numerous leaves, involving physiological trade-offs in the expression of component traits and, possibly, physiological and genetic linkages. This study explores a plant-model-assisted phenotyping approach to dissect the genetic architecture of rice early vigour, applying the Genome Wide Association Study (GWAS) to morphological and NSC measurements, as well as fitted parameters for the functional–structural plant model, Ecomeristem. Leaf size, number, SDW, and source-leaf NSC concentration were measured on a panel of 123 japonica accessions. The data were used to estimate Ecomeristem genotypic parameters driving organ appearance rate, size, and carbon dynamics. GWAS was performed based on 12 221 single-nucleotide polymorphisms (SNP). Twenty-three associations were detected at *P* <1×10^–4^ and 64 at *P* <5×10^–4^. Associations for NSC and model parameters revealed new regions related to early vigour that had greater significance than morphological traits, providing additional information on the genetic control of early vigour. Plant model parameters were used to characterize physiological and genetic trade-offs among component traits. Twelve associations were related to loci for cloned genes, with nine related to organogenesis, plant height, cell size or cell number. The potential use of these associations as markers for breeding is discussed.

## Highlight

Plant-model-assisted phenotyping and metabolomics enable phenotypic and genetic dissection of early vigour traits in a rice diversity panel.

## Introduction

Rice (*O. sativa* L.) is the most important staple food crop worldwide. In 2011, rice crops occupied 164.1M ha [http://faostat.fao.org (accessed 28/01/2013)]. Rice is grown in a variety of environments, covering a wide range of latitudes, altitudes, and hydrologies. This broad adaptation as a species is associated with large genetic and phenotypic diversity (McNally *et al.*, 2009). By providing rapid access to above- and below-ground resources and the ability to compete with weeds, early vigour is a key adaptive behaviour, particularly where levels of mechanization and inputs are low (Asch *et al.*, 1999; Namuco *et al.*, 2009).

Early vigour is commonly estimated by the capacity of seedlings to accumulate shoot biomass rapidly. It is a complex trait, the genetic improvement of which may benefit from its dissection into component traits of lesser genetic complexity. Among such traits, Specific Leaf Area (SLA, cm^2^ g^–1^), tillering, leaf size, leaf appearance, and leaf expansion rates were studied for rice and wheat (Rebetzke *et al.*, 2007; Maydup *et al.*, 2012). Ter Steege *et al.* (2005) reported negative genetic linkages among traits constituting early vigour in wheat, such as leaf size and leaf number. Tisné *et al*. (2008) reported, for *A. thaliana*, a genetic negative linkage between leaf area determination at meristem level and whole-plant leaf production.

Metabolomics was also reported as a relevant phenotyping approach for vigour and growth. Several studies demonstrated that traits for sugar metabolism (NSC or related enzyme activity) were related to pre-flowering growth and were associated with quantitative trait loci (QTL) for rice (Ishimaru *et al.*, 2004, 2007), wheat (Rebetzke *et al.*, 2008), maize (Pelleschi *et al.*, 2006), and *A. thaliana* (Calenge *et al.*, 2006; El-Lithy *et al.*, 2010).

Eco-physiological models can help to dissect phenotypic traits into genotypic and environmental parameters in a heuristic approach. The model can be as simple as a response curve (Reymond *et al.*, 2003) or more complex (Yin *et al.*, 1999; Nakagawa *et al.*, 2005; Quilot *et al.*, 2005). Associating the fitted, genotypic values of model parameters to molecular loci and polymorphisms opens new roles for plant modelling in genetics and breeding. *In silico* simulations can potentially predict QTL effects on the phenotype and explore optimal allele combinations for a given environment (Chenu *et al.*, 2009; Bogard *et al.*, 2014).

Recombinant, bi-parental populations were used for the genetic study of early vigour in cereals (Ter Steege *et al.*, 2005; Zhang *et al.*, 2005; Trachsel *et al.*, 2009). Several genes were identified as controlling early vigour-related traits such as plastochron (Ahn *et al.*, 2002), leaf size (Zhu *et al.*, 2007; Cho *et al.*, 2013), and tillering (Lu *et al.*, 2013; Liang *et al.*, 2014). Rosti *et al.* (2007) studied the effects of a starch deficiency mutant on vegetative growth in rice.

While, so far, plant-model-assisted QTL studies used bi-parental mapping populations, the approach was recently applied by Bogard *et al.* (2014) to a diversity panel of about 200 wheat genotypes to study the genetic control of heading date. The Genome Wide Association Study (GWAS) enables to take advantage of a high density of markers. GWAS associates SNP with the phenotype, mostly using diversity and recombination that evolved naturally. Compared with bi-parental QTL studies, it captures the available allelic diversity on the loci while achieving greater physical resolution due to the smaller linkage disequilibrium (LD) in diversity panels. GWAS is, therefore, an efficient way to dissect the genetic architecture of complex traits and a powerful tool for crop breeding (McNally *et al.*, 2009; Huang *et al.*, 2010; Courtois *et al.*, 2013). Cereals are pioneer crops in this respect (Famoso *et al.*, 2011; Huang *et al.*, 2012; Matsuda *et al*., 2015) although applications in plant breeding are not yet common.

Courtois *et al.* (2013) recently conducted a GWAS study on a rice tropical japonica panel for root growth and morphology traits. Regarding the diversity of above-ground traits constituting early vigour, the same diversity panel of *ca*. 200 tropical japonica accessions was used by Rebolledo *et al.* (2012a). This study confirmed the above mentioned negative correlation between leaf size and number, here represented by leaf appearance rate (syn. development rate, DR). Both traits, however, contributed positively to seedling vigour in terms of rate of shoot biomass accumulation. It was suggested that this trade-off was due to internal competition for assimilates impacting at meristem level (Luquet *et al.*, 2006; Dingkuhn *et al.*, 2007), a key concept for modelling genotypic diversity in growth and phenotypic plasticity. Rebolledo *et al.* (2012b) described the relationship between morphological traits constituting early vigour and NSC in source and sink leaves. Highly vigorous genotypes having small but numerous leaves had low starch but high sucrose concentration in leaves. Genotypes deriving vigour from larger but fewer leaves stored more starch in leaves. Starch reserves can serve as a buffer for stress periods but apparently come at the cost of lesser vigour.

Luquet *et al.* (2012) dissected rice early vigour into component traits using Ecomeristem, a sink driven plant growth model simulating organ appearance and growth rates and their regulation by C resource availability. Model parameters governing processes such as leaf and tiller outgrowth and leaf sizing, estimated for 200 japonica rice accessions, had significant correlations with observed morphological traits and NSC content observed on the same genotypes (Rebolledo *et al.*, 2012a, b), suggesting this model can be useful to analyse the genetic and phenotypic diversity of early vigour traits in rice. Thus, eco-physiological models may serve as tools to analyse the genetic and phenotypic diversity of early vigour traits in rice.

The objective of the present study was to compare early vigour-related traits measured directly (morphology, NSC) or extracted by the Ecomeristem model (fitted process based parameters) and evaluate their added value in support to the phenotypic and genetic study of early vigor diversity in a rice japonica panel. The underlying morphological data, as well as NSC data for a subset of 50 accessions, have previously been reported by Rebolledo *et al.* (2012a, b). The heuristic approach for plant-model-assisted phenotyping with Ecomeristem was described by Luquet *et al.* (2012), and the genomics data obtained with GBS were described by Courtois *et al.* (2013). The hypothesis behind the present study, not previously tested, was that, by better taking into account trait-by-trait interactions and trade-offs among traits constituting early vigour, metabolic and plant-model-assisted phenotyping may add value to conventional phenomics in terms of GWAS results.

## Material and methods

### Plant material

The panel used in this study included 123 accessions, all belonging to the tropical japonica group except three belonging to the temperate japonica group (see Supplementary Table S1 at *JXB* online). All were lines or varieties adapted to rainfed upland ecosystems, except three that were adapted to irrigated flooded systems and four to rainfed-lowland systems. Roughly, half of the panel consisted of improved genetic materials and the other half of traditional varieties or landraces. Seeds of the accessions were obtained either from the Centre de Ressources Biologiques Tropicales de Montpellier or from the International Rice Research Institute (IRRI) gene bank. For each accession, the seeds were produced by single seed descent over two generations in a CIRAD Montpellier greenhouse to ensure that the samples were homogeneous.

### Phenotypic data

Phenotypic data used in the present study included different types of traits (morphological, metabolic, and model parameters) that were reported in three previous publications by the same authors on 202 accessions (Luquet *et al.*, 2012; Rebolledo *et al.*, 2012a, b). The subset of 123 accessions selected for this study was composed in order to dispose of complete data sets for the three types of traits for each accession, and to achieve satisfactory fitting of plant-model parameters to the observed data (details in section on model-based analyses).

A greenhouse experiment with two replications was carried out from 9 February 2009 to 12 May 2009 (late winter and early spring) at CIRAD (Montpellier, France) on the panel described above. Mean daily photosynthetically active radiation (PAR) was 4.6 MJ m^–2^ d^–1^. Air humidity and temperature were regulated by the adiabatic method and were set to 25/22 °C (day/night) and 50/90% air humidity. Seedlings were grown in a germination chamber at 29 °C. When seedlings had reached 3cm in height, five seedlings per pot were transplanted in 1.0 l drained pots (for details, see Rebolledo *et al.*, 2012a). The date of transplanting was variable depending on the accession and its time of germination. Pots were placed on flooded tables with a water depth of 5cm. Plants were thinned to one plant per pot at the 4-leaf stage. Within a replication, each accession was represented by one potted plant in well-watered conditions. Pots contained about 450g (dry weight) of a mixed soil consisting of 20% peat and 80% loamy sandy clay soil.

### Growth and development measurements

For each plant, the date of appearance of leaf 6 on the main stem was noted. Tiller number (NBT), last ligulated leaf length (LLL, cm), appeared leaf number on the main stem, appeared leaf number on the whole plant (NBL), and shoot dry weight (SDW, g plant^–1^) were measured at the end of the experiment, 30–40 d after transplanting depending on the accession and its development rate. Final sampling was not performed at the same date for all accessions because of differences in phenology. Accessions had 8– 9 appeared leaves on the main stem at final sampling. In order to enable comparison among accessions, observations (tiller and leaf number) were normalized by the photo-thermal time experienced by each plant from germination to final sampling, whereas LLL was normalized by its rank (for more detail, see Rebolledo *et al.*, 2012a). Development Rate [DR (°C d^–1^), syn. phyllochron^–1^], defined as number of leaves appeared on the main stem per unit thermal time) and Relative Growth Rate (RGR, g g^–1^ °Cd^–1^) from germination to final sampling were computed as described by Rebolledo *et al.* (2012a).

### Model-based analysis of phenotypes

Ecomeristem is a deterministic crop model simulating plant morphogenesis at organ level at daily time steps using weather and soil hydrological properties as input. The model version used here only simulates the vegetative growth phase of rice. It was described in detail by Luquet *et al*. (2006, 2012). The plant is simulated as an average individual of a population forming a canopy. In the absence of drought, plant organogenetic and morphogenetic processes are driven by sinks (demand for current organ growth, depending on their appearance rate and potential size) and an incremental, aggregate C assimilate source (gross photosynthesis as a function of light interception and a genotypic conversion efficiency, minus maintenance respiration). The resulting daily plant C supply/demand ratio defines Ic, a state affecting growth and development processes depending on genotypic, process-specific parameters. Genotypic parameters describe growth, development, and potential resource acquisition rates, subject to Ic and environment (temperature and PAR in the present study, as water supply was optimal). These parameters are presented in Supplementary Table S1 at *JXB* online. The model was calibrated for each accession using the RGenoud package of the R software (Luquet *et al.*, 2012). For this purpose model simulations of final NBT, NBL, LLL, SDW, and leaf number on the main stem at two dates were fitted to corresponding observations under the environmental conditions encountered by each accession. In contrast to the calibration presented in Luquet *et al.* (2012) (model calibration on average phenotypic data of the two replicates for a given accession), parameters were estimated in the present study by fitting the model conjointly on the two replications for each accession (multi-fitting). Five of the 12 genotypic parameters described in Supplementary Table S1 at *JXB* online were used to decribe each accession: MGR (Meristem Growth Rate, cm); PLASTO (°Cd), SLAP (cm^2^ g^–1^), ICT (unitless), EPSIB (g MJ^–1^ m^2^). Only accessions for which the estimated parameters resulted in less than 15% deviation, on average, between simulated and observed variables were retained. This limited the GWAS study on model parameters to 123 accessions.

As MGR and PLASTO showed a positive correlation in the present study (*r*=0.32, *P* <0.01, with the linear regression PLASTO=44.5+1.16 MGR), similar to previous reports (Luquet *et al.*, 2012, Rebolledo *et al.*, 2012a) on the corresponding morphological data (negative correlation between DR and LLL), the deviation from the linear regression between these two parameters was estimated to quantify the ability of a given accession to combine large leaves (LLL, MGR) and high development rate (DR, or small PLASTO) compared with the observed, average relationship regression line between PLASTO and MGR across all accessions on the panel. This deviation was called DEV_PLASTO_MGR (equation 1). The more negative DEV_PLASTO_MGR, the more the accession is able to combine large leaves and rapid development.

$$DEV\_PLASTO\_MGR=\frac{\left[PLASTO-\left(44.5+1.16MGR\right)\right]\times 100}{\left(44.5+1.16MGR\right)}$$ (1)

With 44.5 and 1.16, the parameters of the linear regression explaining PLASTO from MGR, both model parameters were estimated for each of the 123 accessions. Also the higher the value of DEV_PLASTO_MGR, the lower the ability of an accession to produce large leaves at a high rate, when compared with the average linear regression.

### Sugar concentration analyses

This work analysed NSC content in source leaves for the 123 accessions, thus for a larger panel than the one (50 accesions) described in Rebolledo *et al* (2012a).The two last ligulated leaves (youngest source leaves) were sampled in the morning (before 10.00h) to analyse NSC content: hexoses (glucose GLU and fructose FRU), sucrose (SUC), and starch (STA). Sugar contents were analysed by enzymatic reaction with a spectrophotometric method at 340nm using 96-well micro plates and a Janus pipetting robot as described in Gibon *et al.* (2009). The results are expressed as GLU equivalents per unit dry matter (mg g^–1^) for starch and as soluble sugar per unit dry matter (mg g^–1^) for hexoses and sucrose.

### Statistical analyses

Statistical analyses (ANOVA, Tukey test, Multivariate Analysis) of phenotypic data were performed with statistical software R (http://www.R-project.org) and R package FactoMineR v 1.27 as previously described in Rebolledo *et al.* (2012a, b). Histograms were performed with Sigma Plot software (Version 11, 2008 Systat Software, Inc). Pearson correlations and bar plots were performed using XLSTAT.

### Genotyping and GWAS analysis

Genotyping was conducted at Diversity Arrays Technology Pty Ltd. (DArT P/L, Australia), using a method combining Diversity Arrays Technology (DArT Markers) and a next-generation sequencing technique based on GBS called DArTseq™ (SNPs markers). The original dataset described in Courtois *et al.* (2013) contains 16 664 markers (9 727 DArTs and 6 717 SNPs) genotyped in 167 japonica accessions, with a minor allele frequency (MAF) above 2.5%. To perform GWAS in the 123 tropical japonica subset, SNPs with MAF below 5% within these 123 samples were filtered leaving 12 221 SNPs (7 345 DArTs and 4 876 SNPs). No significant difference was detected between the structure observed in the 167 tropical japonica panel (Courtois *et al.*, 2013) and the one used in this study (data not shown). The number of SNPs available for analysis, resulting in a mean distance of 22.5kb between markers, was sufficient largely to cover the genome because linkage desequilibrium was spanning 150kb, on average, in this population (Courtois *et al.*, 2013). Therefore a distance of ±20kb around each marker, corresponding to the average distance between markers, was used to identify genes of interest. SNPs linked with a strong LD (*r* ^2^ >0.7) were considered as a unique region. To find consistent and significant associations between the genetic markers and phenotypic traits, a mixed model involving population structure as fixed effect and a Kinship matrix (K) as random effect) in Tassel V4.3.6 was used. Population structure was represented by the matrix of accession scores in a Principal Component Analysis (PCA) performed on markers in Tassel using six components to make it consistent with the structure analysis reported for the same tropical japonica panel by Courtois *et al.* (2013). K was generated using the pairwise Identity By State distance approach implemented in Tassel. Plots representing GWAS results (Manhattan and Quantile–Quantile plots) were performed using the package QQman in R. Only associations with *P*-values below the thresholds of 1×10^–4^ (named in this study as significant association) and 5×10^–4^ (named in this study as suggestive association) were considered. The corresponding *q*-values for all traits were computed using the software Q-value v1.0 (Storey and Tibshirani, 2003). Q values measure the False Discovery Rate, which is the proportion of false positives among the tests found to be significant.

## Results

### Phenotypic variation

**Fig. 1.**
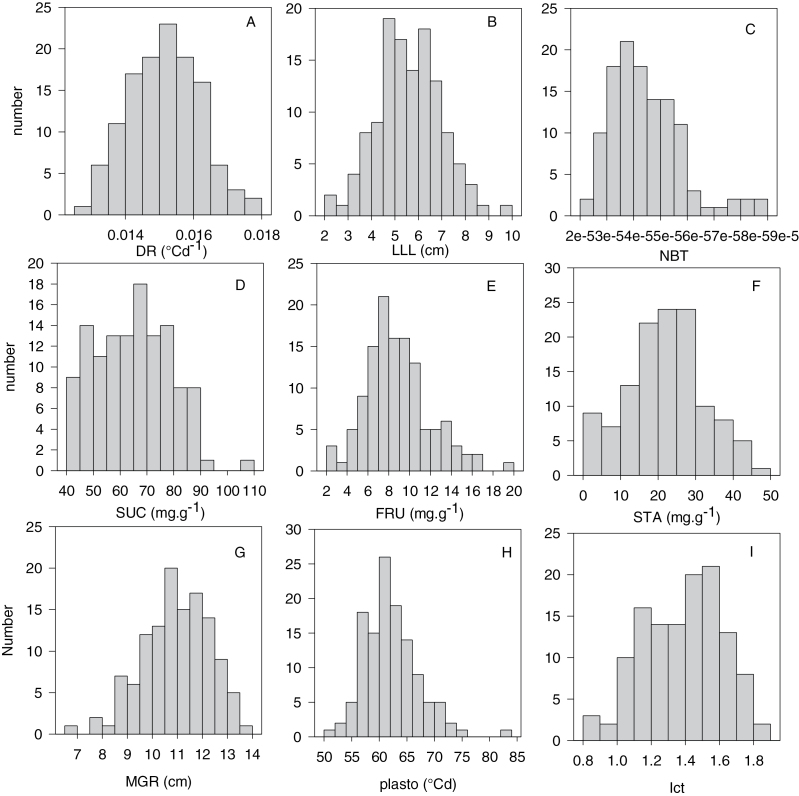
Histogram of the distribution of morphological traits (A, B, C: DR, LLL, NBT), non-structural carbohydrates (D, E, F: SUC, FRU, STA), and ecomeristem model parameters (G, H, I: MGR, PLASTO, ICT) (all defined in Table 1) measured in the japonica rice diversity panel of 123 accessions.

**Fig. 2.**
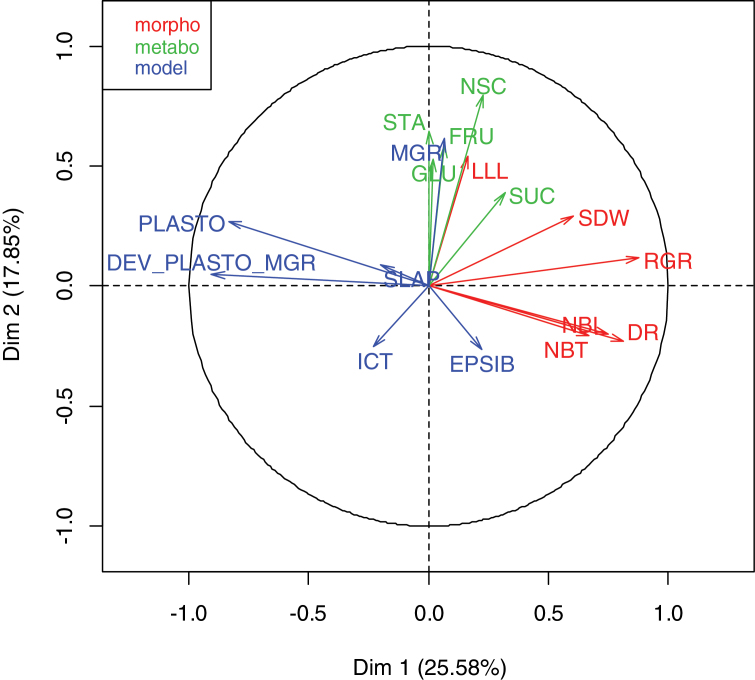
Multiple Factorial Analysis using all the phenotypic variables described in Table 1. Morpho (morphological traits): SDW (shoot dry weight), LLL (Last Ligulated Leaf length), RGR (Relative Growth Rate), DR (Developmental Rate), NBT (Number of Tillers), NBL (Number of Leaves). Metabo (Non-structural carbohydrates concentration in source leaves related traits): STA (starch), SUC (sucrose), FRU (fructose), GLU (glucose), NSC (total non-structural carbohydrates). Model (Ecomeristem model parameters): PLASTO (Phyllochron), DEV_PLASTO_MGR (% deviation from the linear regression between PLASTO and MGR), SLAP (Slope Parameter of the negative logarithmic equation computing SLA for successive leaf ranks), MGR (Meristem Growth Rate), ICT (IC Threshold enabling tiller outgrowth), EPSIB (Light conversion efficiency).

Figure 1 shows the distribution of a wide range of values found in the diversity panel for key traits. The observed variables DR, LLL, and NBT (Fig. 1A, B, C, respectively) were normally distributed. Among the NSC concentrations in source leaves (Fig. 1D, E, F), SUC showed a comparatively narrow band of relative variation (factor 2), whereas FRU concentration varied from 2 to 20mg g^–1^ (factor 5) and starch from 0 to 50mg g^–1^ on a dry weight basis. The distributions of genotypic model parameters (Fig. 1G, H, I) were normal with a generally smaller relative range of variation: MGR from 7 to 14cm, PLASTO from 50 to 85 °Cd, and ICT from 0.8 to 1.8. The distribution of other traits can be found in Supplementary Fig. S1 at *JXB* online. To identify the relation between traits, a Multiple Factorial Analysis (MFA, Fig. 2) was performed using the variables listed in Table 1. Supplementary Table S2 at JXB online shows the corresponding matrix of correlation. Two dimensions explained 43% of the phenotypic diversity (Fig. 2). Dimension 1 (explaining 26%) combined effects of RGR and DR (positive) and DEV_PLASTO_MGR and PLASTO (negative). RGR, the relative growth rate which was also associated with SDW, clustered with variables related to organ number (DR, NBL, and NBT). The correlations between RGR and SDW versus variables related to organ number were significant (*P* <0.01) (see Supplementary Table S2 at *JXB* online). Regarding the NSC traits, SUC was the only variable having an effect in Dimension 1 (positive). This was associated with a significant positive correlation (*P* <0.01) between SUC and both DR and SDW (see Supplementary Table S2 at *JXB* online).

**Table 1.** Traits studied on the diversity panel of 123 genotypes

Trait	Definition
**Morphological**	
NBL	Green leaf number on the plant
NBT	Tiller number per plant
LLL	Last ligulated leaf length (cm)
DR	Development rate (°Cd^–1^)
RGR	Relative Growth Rate (g g^–1^ °Cd^–1^)
SDW	Shoot Dry Weight per plant (normalized by the photo thermal time)
**Model parameters**	
EPSIB	Light conversion efficiency (g MJ^–1^ m^2^ d)
ICT	Ic threshold enabling tiller outgrowth
MGR	Meristem Growth Rate, leaf length increment between two consecutive leaves (cm)
PLASTO	Plastochron or phyllochron (°Cd)
SLAP	slope parameter of the negative logarithmic equation computing SLA for successive leaf ranks (cm^2^ g^–1^)
DEV_PLASTO_MGR	% of deviation from the linear regression between PLASTO and MGR (computed as PLASTO-
**Non-structural sugar concentration**	
GLU	[Glucose] in youngest source leaves (mg g^–1^ dw)
FRU	[Fructose] in youngest source leaves (mg g^–1^ dw)
SUC	[Sucrose] in youngest source leaves (mg g^–1^ dw)
STA	[Starch] in youngest source leaves (mg g^–1^ dw)
NSC	[Non Structural Carbohydrate] (GLU+FRU+SUC+STA) in youngest source leaves (mg g^–1^ dw)

Dimension 2 (explaining 18%) was mainly composed of sugar-related traits (STA, NSC, GLU, FRU; all positive) and organ size (MGR as model parameter, LLL for measured trait; both positive). This is confirmed in Supplementary Table S2 at *JXB* online by positive, significant correlations between FRU and LLL, and between NSC and MGR. SDW was more explained by organ number (Dimension 1) than by organ size-related traits (Dimension 2). In general, few significant correlations were found between sugar-related traits and other types of traits.

### Association mapping

**Fig. 3.**
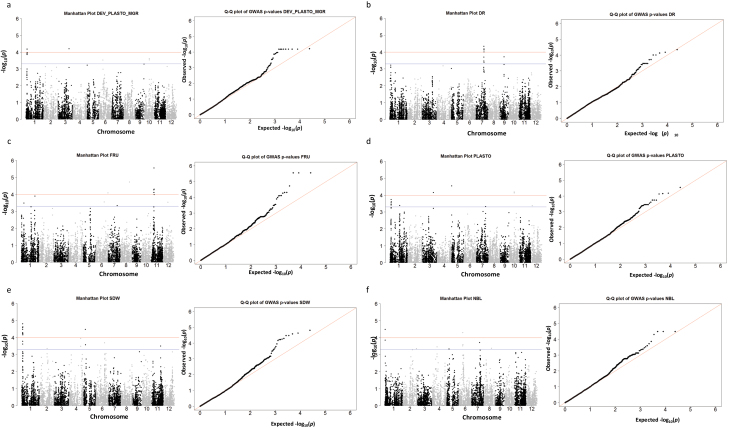
QQ plots and Manhattan plots for traits associated to markers with the highest level of significance: FRU, PLASTO, DEV_PLASTO_MGR, SDW, NBL, DR. In the Manhattan plots the red line indicates the threshold for significant SNP association (*P* <1×10^–4^) and the blue line indicates the threshold for suggestive SNP association (*P* <5×10^–4^).

Figure 3 presents Quantile–Quantile (QQ) plots and Manhattan plots for the traits with the most significant detection levels (FRU, PLASTO, DEV_PLASTO_MGR, SDW, NBL, DR). Supplementary Fig. S2 at *JXB* online shows QQ plots and Manhattan plots for the other traits. A total of 23 QTL were identified that were significantly associated with either morphological traits, sugar-related traits or model parameters at *P* <1×10^–4^. For approximately two-thirds of the significant associations, the *P*-values corresponded to *q*-values below 0.10 (Table 2). Five QTL were significantly associated with morphological variables, eight with model parameters and seven with metabolic traits, the latter mainly on chromosome (chr) 11 and related to FRU and GLU in source leaves.

**Table 2.** QTL identified with significant association to phenotypic traits (*P*-value <1×10^–4^)

QTL name	Chr	SNP position	LD region	Trait	MAF	P-value	*q*-value	Marker R2	No. of reported genes in ±20kb
1	1	735758	735758–754061	NBL, NBT	0.11	3.25E-05	0.09	0.13	8
2	1	859614	859614	NBT	0.11	5.13E-05	0.09	0.13	7
3	1	2270163	2270163–2274710	SDW	0.29	5.11E-05	0.08	0.13	5
4	1	2831348	2831348–2845870	DEV_PLASTO_MGR	0.28	6.56E-05	0.09	0.12	6
5	3	27040569	27040569	DEV_PLASTO_MGR, PLASTO	0.15	6.22E-05	0.09	0.12	3
6	5	62353	62353	PLASTO	0.23	2.83E-05	0.22	0.12	3
7	5	3868146	3868146	SDW	0.32	3.31E-05	0.08	0.13	2
8	6	9355224	9355224	DEV_PLASTO_MGR, RGR	0.46	5.18E-05	0.09	0.12	5
9	6	9653998	9653998	NBL	0.37	4.99E-05	0.13	0.12	3
10	6	22069867	22069867	MGR	0.18	5.41E-05	0.44	0.12	2
11	6	30484095	30484095	FRU, GLU	0.15	8.11E-05	0.09	0.11	5
12	7	19079282	19079282	DR	0.50	4.53E-05	0.23	0.12	10
13	7	19347738	19347738–19475740	DR	0.20	9.85E-05	0.23	0.11	2
14	8	22122426	22122426	FRU	0.06	1.88E-05	0.05	0.13	6
15	9	14409525	14409525–14455030	LLL, MGR	0.07	9.12E-05	0.30	0.10	5
16	10	11705724	11705724	DEV_PLASTO_MGR	0.23	6.54E-05	0.09	0.11	4
17	10	11712638	11712638	PLASTO	0.25	7.42E-05	0.22	0.11	2
18	11	3638283	3638283–3666205	NBT	0.46	3.68E-05	0.09	0.12	6
19	11	7272543	7272543	FRU	0.15	7.79E-05	0.09	0.11	2
20	11	7329181	7329181	FRU	0.12	5.36E-05	0.08	0.12	4
21	11	7538208	7538208	FRU	0.07	7.65E-05	0.09	0.11	3
22	11	7603153	7603153–7609931	FRU, GLU	0.10	2.79E-06	0.01	0.15	4
23	11	7693392	7693392–7693395	FRU	0.09	4.86E-05	0.08	0.12	5

### QTL, trait and gene associations

A total of 120 genes for the significant QTL (*P* <1×10^–4^) were found within ±20kb regions. Three QTL for the model parameter (DEV_PLASTO_MGR), the morphological variable (DR), and the metabolic variable (FRU_GLU) were associated with cloned genes having known phenotypic functions on panicle development, floral meristem size or meiosis (Table 2). A total of 314 genes for all the suggested QTL (*P* <5×10^–4^) reported in Supplementary Table S3 at *JXB* online were found within the ±20kb regions. Nine suggestive QTL (*P* <5×10^–4^), related either to early vigour (SDW or RGR) or its component morphological variables (LLL), model parameters (SLAp, MGR, DEV_PLASTO_MGR) or metabolic traits (NSC), were associated with cloned genes with a known function on vegetative organs (seedling vigour, cell production, and cell size) or reproductive organs (anther dehiscence) (Table 3).

**Table 3.** Colocation of markers with genes already identified or published

Measured trait	Chr	SNP position	Locus name	Annotation	Gene name	Gene function	Reference
**Cloned genes in suggestive associated markers (*P* <5×10** ^**–4**^)							
**LLL**	1	34167105	LOC_Os01g59120	Cyclin, putative, expressed	Cyclin B1;1 (CycB1;1)	Endosperm formation. Embryo size.	(Guo *et al.*, 2010)
**SDW**	2	18637296	LOC_Os02g31140	Major ampullate spidroin 2-2, putative, expressed	Curly flag leaf1 (cfl1)	Curly leaf. Leaf cuticle development.	(Wu *et al.*, 2011)
**RGR**	2	32101207	LOC_Os02g52480	Cyclin-dependent kinase inhibitor, putative, expressed	Kip-related protein1 (KRP1)	Reduced cell production and increased cell size. Seed maturation	Barrôco *et al.* (2006)
**RGR**	2	32101207	LOC_Os02g52490	Expressed protein	SG1-Like protein 1 (SGL1)	Culm and rachis internode elongation. Seed development. Cell proliferation. Leaf angle. Brassinoide sensitivity.	Nakagawa *et al.* (2012)
**MGR**	3	32655537	LOC_Os03g57240	ZOS3-19 - C2H2 zinc finger protein, expressed	Drought and salt tolerance (DST)	Drought and salinity tolerance, Stomatal control	(Huang *et al.*, 2009)
**NSC**	5	1445349	LOC_Os05g03430	ATSIZ1/SIZ1, putative, expressed	SUMO E3 ligase 1 (siz1)	Anther dehiscence	Thangasamy *et al.* (2011)
**SLAP**	7	4334575	LOC_Os07g08420	bZIP transcription factor domain containing protein, expressed	bZIP transcription factor 58 (OsbZIP58)	Seed development, Seed starch content	(Wang *et al.*, 2013)
**DEV_PLASTO_ MGR**	9	21275388	LOC_Os09g36910	bZIP transcription factor domain containing protein, expressed	FD (OsFD1)	Flowering time	Taoka *et al.* (2011)
**SDW**	11	22526446	LOC_Os11g37970	WIP5–Wound-induced protein precursor, expressed	Pathogenesis- related 4 (OsPR4)	Drought tolerance	(Wang *et al.*, 2011)
**Cloned genes in significantly associated markers (*P* <1×10** ^**–4**^)							
**DEV_PLASTO_ MGR**	6	9355224	LOC_Os06g16370	CCT/B-box zinc finger protein, `putative, expressed	Heading date 1 (Hd1)	Panicle development, Heading Date	Weng *et al.* (2014)
**FRU-GLU**	6	30484095	LOC_Os06g50340	Receptor protein kinase, CLAVATA1 precursor putative, expressed	Floral Organ Number1 (fon 1)	Floral organ number, Floral meristem size	Suzaki *et al.* (2004)
**DR**	7	19347738	LOC_Os07g32480	Mitotic checkpoint serine/ threonine-protein kinase BUB1,putative, expressed	Bub1-Related Kinase 1 (BRK1)	Meiosis	(Wang *et al.*, 2012)

Two QTL were significantly associated with SDW on chr 1 and chr 5 and a total of eight QTL were significantly associated with morphological component traits (Table 2). NBL was significantly associated with a QTL on both chr 6 and another on chr 1 (Table 2). For NBL, a strong LD (*r* ^2^ >0.7) on chr 1 was observed between two markers (735 758 and 754 061) (Table 2). 109 accessions carrying the T allele in position 735 758 and the A allele in position 754 061 had a lower leaf number than 14 accessions having the A allele in position 735 758 and the G allele in position 754 061 (see Supplementary Fig. S3A, B at *JXB* online). However, no gene with known function was reported within this QTL region. DR was significantly associated at QTL12 on chr 7 at position 19 079 282 (Table 2) and colocated with the Bub1-Related Kinase 1 (BRK1) gene involved in meiosis (19 012bp distance) (Table 3) (Wang *et al.*, 2012). For this QTL, the accessions having the A allele showed lower DR values (see Supplementary Fig. S3F at *JXB* online) than accessions having the T allele. Only one QTL was significantly associated with LLL on chr 9 (Table 2). LLL, DR or NBT were never associated in the same QTL region. Some of the suggestive QTL (*P* <5×10–4) (see Supplementary Table S3 at *JXB* online) were also associated with genes with known function (Table 3): a QTL associated with RGR on chr 2 colocated with the KRP1 gene, increasing cell size and reducing cell production (Barroco *et al.*, 2006), and with the SGL1 gene controlling grain elongation via a SG1-Like Protein1 (Nakagawa *et al.*, 2012).

A total of seven QTL were significantly associated with GLU and FRU only (Table 2) and were never associated together with morphological variables or model parameters. GLU and FRU were significantly associated together with QTL11 (Table 2). At this locus, the 18 accessions carrying allele A showed higher hexose concentrations (11.17mg g^–1^ for FRU and 17.3mg g^–1^ for GLU) compared with accessions with allele G (see Supplementary Fig. S3G at *JXB* online). Significant associations for GLU and FRU were also observed on chr 6 at position 9 355 224 (Table 2). The FLO1 gene controlling a protein kinase, CLAVATA1 (Suzaki *et al.*, 2004), involved in floral meristem size and floral organ number, was 14 050bp distant from this marker. FRU was also significantly associated with QTL19, 14, and 22 on chr 11 (Table 2). No gene with a known function was previously reported in this region. Only one suggested association was observed for NSC on chr 5 (see Supplementary Table S3 at *JXB* online); it was within the 20kb region from the Rice S1Z1 (SUMO E3) ligase 1 protein gene that controls anther dehiscence (Thangasamy *et al.*, 2011) (Table 3).

Among the model parameters, eight significant QTL were detected, mainly for (DEV_PLASTO_MGR) (Table 2). DEV_PLASTO_MGR was significantly associated with four markers on chr 1, 3, 6, and 10; PLASTO was significantly associated with markers on chr 3, 5, and 10; and MGR was significantly associated with two markers on chr 6 and 9 (Table 2). Regarding PLASTO, the strongest association was observed on chr 5 (Fig. 3) at position 62 353. Accessions with the T allele showed higher PLASTO, thus lower leaf appearance rates, than accessions with the C allele at this locus (see Supplementary Fig. S3C at *JXB* online). PLASTO colocalized with NSC on chr 7 at position 22 559 297 but this association had *P* <5×10^–4^ (see Supplementary Table S3 at *JXB* online). However, the phenotypic effects of these traits were located on different MFA dimensions (see Supplementary Fig. S3D, E at *JXB* online). Allele A had a negative effect on NSC (105mg g^–1^ versus 120mg g^–1^) and a positive effect on PLASTO (62.79 °Cd versus 59.78 °Cd). No cloned gene was positioned near this marker.

Model parameters significantly colocalized with morphological traits for only two QTL: on chr 9 (QTL15: LLL and MGR, both related to leaf size and positively correlated; see Supplementary Table S3 at *JXB* online) and on chr 6 at position 9 355 224 (QTL8: RGR and DEV_PLASTO_MGR) (Table 2), with a similar level of significance. The latter co-detection for QTL8 suggests that high RGR is associated with the plant’s ability to combine both high leaf production rate and large leaf size (equation 1). This QTL8 was located at 18 865bp distance from the widely known HD1 gene controlling heading date (Taoka *et al.*, 2011) and panicle size (Weng *et al.*, 2014), via a CCT/B-box zinc finger protein (Table 3). In this study, 57 accessions with the T allele at QTL8 showed lower DEV_PLASTO_MGR values (0.1) and higher RGR values (1.014) compared with accessions with the C allele (see Supplementary Fig. S3D, E at *JXB* online). The wide distribution observed for DEV_PLASTO_MGR within each group carrying each allele explained the high standard deviation observed (data not shown).

## Discussion

In spite of the considerable knowledge on traits conferring early vigour, there is little evidence on the added value of such secondary traits when explicitly included in rice breeding programmes. This may be explained by the lack of knowledge on: (i) the compensation among traits, (ii) their genotypic diversity and genetic architecture, and (iii) their behaviour in different environments. The present study aimed to combine morphological, metabolic, and model parameters for the phenotypic and genetic dissection of early vigour. It was expected from ‘hidden’ phenotypic markers (NSC variables and process-based model parameters heuristically estimated) to provide further insight into underlying trait–trait correlations and trade-offs. The approach was tested on a japonica rice diversity panel by performing GWAS for these traits.

### Trait associations and contributions to early vigour

The three types of traits addressed in the present study were confirmed to contribute to early vigour, characterized by biomass accumulation (SDW). SDW was significantly correlated with morphological traits (LLL, NBT, NBL, DR), model parameters (MGR, PLASTO, ICT, DEV_PLASTO_MGR), and metabolic traits (SUC). This confirmed the results reported by Luquet *et al.* (2012) for model parameters and Rebolledo *et al.* (2012a, b) for metabolic traits and morphological traits in a subset of the japonica panel (50 accessions). Using MFA, the relative contribution of the various traits to SDW could be evaluated in this study. Development rate (DR, positive) or its reciprocal model parameter PLASTO (negative) contributed to early vigour. The strongest contribution to vigour was observed, however, for the derived model parameter DEV_PLASTO_MGR, expressing the combined ability of a genotype to produce both large and numerous leaves on a given culm. Plant leaf number (NBL) and tiller number (NBT) contributed similarly to vigour, whereas leaf length (LLL) and its functional model equivalent MGR alone showed a small contribution.

Among the metabolic traits, leaf sucrose content showed the highest (positive) contribution to vigour whereas starch and hexose contents contributed strongly (positively) to leaf size (observed LLL and MGR model parameter) but did not have a significant effect on vigour.

It therefore appears that early vigour was favoured first, by a high development rate associated with a high sucrose concentration in source leaves, and was further increased by large leaves which were associated with high starch and hexose concentrations in the source leaves. This was translated in the model by a combination of low PLASTO and high MGR values. In the model, effects on the phenotype of PLASTO (affecting leaf appearance rate), MGR (affecting leaf size), and ICT (affecting tillering) are subject to compensatory responses due to assimilate limitation. It is interesting that PLASTO and MGR, which act at the culm scale, together had a dominant impact on early vigour, whereas ICT, which acts on tiller number at the plant scale, had a comparatively small effect. Since these parameter effects were evaluated on the basis of parameter fitting to each of the accessions, they reflect an observed pattern across the panel.

### GWAS for traits contributing to early vigour

In general, traits targeted in the present study were associated with numerous markers with significant but not very high effects, confirming the evidence that early vigour is controlled by many QTL with small effect. This was also the case in other studies on other complex rice seedling traits such as root architecture (Courtois *et al.*, 2013; Sandhu *et al*., 2015). The population used here for GWAS was small (123) compared with the diversity panels commonly used for such studies (>200 accessions: Huang *et al.*, 2010; Yang et al, 2014), which probably limited the attainable levels of significance for the associations. However, this study dealt with physiological traits more difficult to phenotype than plant height or grain weight (Huang *et al.*, 2010). Conclusive GWAS using small populations have previously been reported: betweeen 87 and 97 accessions for the components of a rice panel (Zhao *et al.*, 2011) or 95 for *Arabidopsis* (Aranzana *et al.*, 2005).

A total of 23 QTL were identified having significant associations with *P* <1×10^–4^ (Table 2). Colocalizations for different traits were rare (maximum 2). QTL for all types of traits were identified, but three model parameters (MGR, PLASTO and the derived, aggregated parameter DEV_PLASTO_MGR) and leaf fructose concentration (FRU) gave the strongest associations. This suggests that component traits derived heuristically by the Ecomeristem model or NSC measurement strengthened the analysis of early vigour. In fact, only one QTL on chr 1 (Table 2) was significantly associated with SDW, the variable defining early vigour and being interpreted here as the complex trait functionally composed of the component traits. This QTL did not colocalize with any of the other traits and could not be associated with any published, cloned gene (Table 3). It is thus an interesting marker that might be associated with a component trait of early vigour that was not measured in this study.

### Relevance of model-based and metabolic traits compared with morphological traits

Rebolledo *et al.* (2012a) suggested that DR is a key component trait explaining the genotypic variation of early vigour, through rapid deployment of organs and thus, sinks. This study indicated that genetic control of DR is related to two QTL having *P* <1×10^–4^. This result potentially opens perspectives for marker-assisted selection for DR in breeding. Two recent field studies, focusing mainly on morphological traits, evaluated biparental japonica×indica crosses (Zhou *et al.*, 2007; Sandhu *et al*., 2015) and reported QTL for early vigour or seedling vigour. Several QTL identified in the present study by either morphological or model parameters were situated near genes controlling seed size (Table 3) which may then contribute to seedling vigour (Wang *et al.*, 2010).

The negative correlation between leaf size and number (LLL versus DR) can be interpreted as a trade-off between two traits that contribute to early vigour, because both increase the sink strenght for a finite assimilate resource (Luquet *et al.*, 2012; Rebolledo *et al.*, 2012a). GWAS results did not show any shared genomic association of these traits, nor did the related model parameters MGR and PLASTO. Due to the functional similarity between MGR and LLL, they shared QTL15 on chr 9. However, the aggregated model parameter DEV_PLASTO_MGR combining leaf size and number effets was associated with three QTL that colocalized neither with MGR nor with PLASTO. It was associated, together with RGR, with QTL6. This QTL colocalized with the HD1 gene (Table 3), related to heading date, plant height, and seed weight (Lu *et al.*, 2007). Other (non-cloned) genes were also reported in this region (Table 3). Accordingly, this region is promising to search for genes related to early vigour combining large and numerous leaves. It would also be of interest to determine if the effects of QTL6 on earliness, plant height, seed weight, and early vigour (or RGR) rely on the same gene or a cluster of complementary genes, as sometimes observed (Zhang *et al.*, 2012). If this is the case, the trade-off between leaf size and number would not only be physiological but might also represent a physical linkage between different but complementary genes, as suported by Tisné *et al*. (2008) and Ter Steege *et al.* (2005), for *A. thaliana* and wheat, respectively, suggesting a strong negative genetic linkage among these traits, constraining their co-selection. Identifying QTL controlling specifically or conjointly leaf size and leaf deployment rate, as in the present study, is a crucial step to overcome such genetic linkages for improving a complex trait such as early vigour.

Metabolic traits were previously reported in rice to participate in the expression of complex traits such as early vigour (Rebolledo *et al.*, 2012a) and grain yield (Ishimaru *et al.*, 2007). One difficulty for the genetic dissection of this type of trait is that tissue concentrations fluctuate rapidly in response to environment (Rebolledo *et al.*, 2012b) and plant development (He *et al.*, 2005; Luquet *et al.*, 2008). In this study, QTL were associated with sugar concentrations, mainly on chr 11 for hexoses (GLU, FRU) in source leaves. This region was narrow (position 7 603 153 to 7 609 931bp) and different from that associated with total NSC concentration. On chr 6, FRU and GLU were associated with the cloned gene Floral Organ Number 1 controlling meristem size (Table 3). Hexose concentration was mainly correlated with LLL (Fig. 2), suggesting that pools of different sugars might have different physiological roles and also depend on organ type and age (Sulpice *et al.*, 2009; Rebolledo *et al.*, 2012a). One QTL related to total NSC in the present study was associated with the PLASTO model parameter, and allelic effects were in the same direction for both traits. This is in agreement with Rebolledo *et al.* (2012a) who reported that high PLASTO is correlated with high leaf starch concentration. Physiologically, this was interpreted as high leaf starch concentration resulting from low sink activity for assimilates in the plant and, consequently, low vigour. However, neither starch nor sucrose concentrations were significantly associated with any genetic marker in the present study. Further studies using a larger panel and more SNP markers might elucidate QTL controlling leaf sucrose and starch concentration with greater power of trait association (Korte and Farlow, 2013).

### Added value from model-assisted phenotyping

The use of modelling to support genetic studies has already proved its potential in the case of bi-parental populations, based on less-complex models and applied to less-complex traits (Yin *et al.*, 1999; Reymond *et al.*, 2003). One recent study combined GWAS with model-assisted phenotyping to dissect the physiological and genetic architecture of a complex trait (heading date in wheat: Bogard *et al.*, 2014). The reasons for using heuristic applications of models in phenotyping are several (Dingkuhn *et al.*, 2005). For example, the genetic variation of a physiological process or response trait may be difficult to measure (‘hidden trait’) because it is masked by environmental noise and trait×trait interactions, or it constitutes a response to environment itself (Reymond *et al.*, 2003), requiring a model to extract the genotypic information. Once model parameters related to QTL and allele effects, the physiological model should be used subsequently to predict QTL effects on the expression of a complex trait of recombinants and in a different environment, by means of *in silico* extrapolation (Chenu *et al.*, 2009). Such predictions can only be accurate if the genetic linkages and pleiotropic effects among the simulated traits are accounted for in the modelling process, and if physiological interactions with other traits are simulated (Cooper *et al.*, 2014). With this respect, the present study provides further demonstration of the added value of modeling, and provides further validation of the physiological and genetic meaning of Ecomeristem model parameters, as previously suggested by Luquet *et al*. (2012). More research is needed to attain the skill to combine genetic studies and crop modelling to build *in silic*o genetics and physiology-based ideotypes accurately. Based on direct measurements and on the heuristic estimation of model parameters, revealed new component traits of early vigour, involving phenotypic trade-offs and controlled by different QTL. Once validated through fine mapping, expression analysis, and/or development of NIL populations carrying combinations of alleles at QTL controlling leaf size and leaf development, the results might help breeders to develop plants having increased early vigour.

## Conclusions

This study suggested that the complex genetic architecture of rice early vigour is controlled by several genomic regions with small but significant effects on the phenotype. It confirmed a physiological trade-off between leaf size and development rate and provided insight into their individual and common genetic control. Early vigour might be improved by combining both large leaves and high leaf deployment rate or development rate. Metabolic traits, mainly hexose content in source leaves, and Ecomeristem model parameters, in particular those controlling leaf size and leaf appearance rate, helped to understand the genetic and physiological architecture of early vigour. As a next step, Ecomeristem can be used to predict QTL effects on rice seedling performance depending on the environment. The results should be extended and validated for a wider range of genetic diversity and using a higher molecular marker density. Marker effects should be also validated in the field.

## Supplementary data

Supplementary data can be found at *JXB* online.

Supplementary Table S1. A detailed description of the 123 accessions (name, group, system, and type).

Supplementary Table S2. The correlation matrix for all the variables studied.

Supplementary Table S3. All the suggestive QTL detected at *P*-values <5×10^–4^.

Supplementary Fig. S1. The distribution of other variables studied.

Supplementary Fig. S2. shows the Manhattan and Q-Q Plot for other variables studied.

Supplementary Fig. S3. The allele effects for significant QTl.

Supplementary Data
