# Supplementary material for: Phenotypic and genetic dissection of component traits for early vigour in rice using plant growth modelling, sugar content analyses and association mapping
Source: J Exp Bot. 2015 May 28;66(18):5555–66. doi: 10.1093/jxb/erv258 (PMC4585419; doi:10.1093/jxb/erv258)
Supplement: Supplementary Data [file supp_66_18_5555__index.html]

Phenotypic and genetic dissection of component traits for early vigour in rice using plant growth modelling, sugar content analyses and association mapping — Phenotypic and genetic dissection of component traits for early vigour in rice using plant growth modelling, sugar content analyses and association mapping — Supplementary Data 

# Phenotypic and genetic dissection of component traits for early vigour in rice using plant growth modelling, sugar content analyses and association mapping

## Supplementary Data

Data files

- Supplementary Data - Supplementary Data
