# Supplementary material for: Phenotypic and genetic dissection of component traits for early vigour in rice using plant growth modelling, sugar content analyses and association mapping
Source: J Exp Bot. 2015 May 28;66(18):5555–66. doi: 10.1093/jxb/erv258 (PMC4585419; doi:10.1093/jxb/erv258)
Supplement: Supplementary Data [file supp_erv258_jexbot142489_file001.pdf]

Phenotypic and genetic dissection of component traits for early vigor in rice using plant growth modelling, sugar content analyses and association mapping

Rebolledo M.C., Dingkuhn M, Courtois B, Gibon Y. Clément-Vidal A., Cruz D.F, Duitama J., Lorieux M. and Luquet D.

Supplementary Figure legends:

Fig. S1 : Histogram of distribution of other morphological variables and model parameters used for GWAS. Acronyms are explained in Table 2 of the manuscript.

Fig.S2 : Manhattan and Quantile – Quantile Plots for SUC, STA, SLAP, NSC, LLL, ICT, GLU, MGR, NBT and EPSIB. In the Manhattan plots the red line indicates the threshold for significant SNP association ($P<1.10^{-4}$) and the blue line indicates the threshold for suggestive SNP association ($P<5.10^{-4}$).

Fig. S3: Allele effects at markers associated with non-reported (A-C) and reported (D-G) genes. Phenotypic traits are described in Table 2 and QTL are described in Table 3.

Table S1 : List of the 123 accessions: name, genetic group (Trjap: tropical japonica, Tejap: temperate japonica), agro-ecosystem (system: U=upland, RL=rain-fed lowland, I=irrigated) and type (T=traditional, I= improved, No available information (No Inf).

Code	name	group	System	type	Code	name	group	System	type
4	AZUCENA	Trjap	U	T					
7	BULU PANDAK	Trjap	RL	T	353	IRAT 364	Trjap	U	I
13	GIZA 171	Tejap	I	I	354	IRAT 366	Trjap	U	I
14	IAC 165	Trjap	U	I	356	JAO HAW	Trjap	U	T
23	NIPPONBARE	Tejap	I	I	357	JIMBRUK JOLOWORO	Trjap	U	T
250	62667	Trjap	U	T	358	JUMALI	Trjap	U	T
252	ARAGUAIA	Trjap	U	I	360	KAKANI 2	Trjap	U	T
253	ARIAS	Trjap	U	T	361	KANIRANGA	Trjap	U	T
254	ARROZ CEBADA	Trjap	U	T	362	KARASUKARA SURANKASU	Trjap	U	No Inf
255	BABER	Trjap	U	T	363	KEDAYAN	Trjap	U	T
256	BAGANAN ASALAO	Trjap	U	T	365	KENDINGA 5 H	Trjap	U	T
257	BAKUNG H	Trjap	U	T	367	KETAN KONIR	Trjap	RL	T
259	BENGALY VAKARINA	Trjap	U	T	369	KETAN MENAH	Trjap	U	T
260	BICO BRANCO	Trjap	U	T	371	KHAO KAP XANG	Trjap	U	T
261	BINULAWAN	Trjap	U	T	372	KINANDANG PATONG	Trjap	U	T
265	CAAWA/FORTUNA 6	Trjap	U	I	373	KOMOJAMANITRA	Trjap	U	T
268	CANELA DE FERRO	Trjap	U	T	374	KU 115	Trjap	U	I
269	CHA LOY OE	Trjap	U	T	375	KUROKA	Tejap	I	I
270	CHA PHU MA	Trjap	U	T	377	LAMBAYQUE 1	Trjap	U	T
273	CICIH BETON	Trjap	No Inf	T	379	LUDAN	Trjap	U	T
275	CIRAD 358	Trjap	U	I	381	MAINTIMOLOTSY 1226	Trjap	U	T
276	CIRAD 392	Trjap	U	I	382	MALAGKIT PIRURUTONG	Trjap	U	T
277	CIRAD 394	Trjap	U	I	385	MARAVILHA	Trjap	U	I
279	CIRAD 403	Trjap	U	I	387	MITSANGANAHIJERY	Trjap	U	T
281	CIRAD 488	Trjap	U	I	388	MOLOK	Trjap	RL	T
282	CNA-7\BO\1\1>33-13-6-1	Trjap	U	I	389	NABESHI	Trjap	No Inf	No Inf
283	COLOMBIA 1	Trjap	U	I	390	NEP HOA VANG	Trjap	U	T
284	CT13582-15-5-M	Trjap	U	I	391	NHTA 10	Trjap	U	T
291	CURINCA	Trjap	U	I	392	NHTA 5	Trjap	U	T
292	DAM	Trjap	U	T	397	ORYZICA SABANA 6	Trjap	U	I
294	DAVAO	Trjap	U	T	398	OS 4	Trjap	U	T
296	DINORADO	Trjap	U	T	400	P5589-1-1-3-P	Trjap	U	I
297	DOURADO AGULHA	Trjap	U	T	401	PACHOLINHA	Trjap	U	T
298	DOURADO PRECOCE	Trjap	U	T	404	PADI KASALLE	Trjap	No Inf	T
301	FOHISOMOTRA	Trjap	U	T	410	PCT11\0\0\2,BO\1>55-1-3-1	Trjap	U	I
303	GANIGI	Trjap	U	T	412	PCT4\SA\4\1>1076-2-4-1-5	Trjap	U	I
304	GEMJYA JYANAM	Trjap	No Inf	T	413	PEH PI NUO	Trjap	No Inf	T
**Code**	**name**	**group**	**System**	**type**	**Code**	**name**	**group**	**System**	**type**

305	GOGO	Trjap	U	T	417	RATHAL	Trjap	U	T
306	GOGO LEMPUK	Trjap	U	T	418	REKET MAUN	Trjap	RL	T
307	GOGO LEMPAK	Trjap	U	T	419	RT 1031-69	Trjap	U	T
308	GOMPA 2	Trjap	U	T	422	SENG	Trjap	U	T
310	GRAZI	Trjap	U	T	428	TREMBESE	Trjap	U	T
311	GUARANI	Trjap	U	I	429	TRES MESES	Trjap	U	T
312	GUNDIL KUNING	Trjap	U	T	430	TSIPALA 89	Trjap	U	T
319	IAC 25	Trjap	U	I	432	VARY LAVA 90	Trjap	U	T
321	IDSA 77	Trjap	U	I	433	VARY LAVA DE BETAFO	Trjap	U	T
322	IGUAPE CATETO	Trjap	U	T	437	VARY SOMOTRA SIHANAKA	Trjap	U	T
325	IR60080-46A	Trjap	U	I	438	WAB 56-125	Trjap	U	I
327	IR63380-08	Trjap	U	I	439	WAB 56-50	Trjap	U	I
328	IR63372-08	Trjap	U	I	440	WAB706-3-4-K-KB-1	Trjap	U	I
331	IR65907-188-1-B	Trjap	U	I	443	YANCAOUSSA	Trjap	U	T
334	IR68704-145-1-1-B	Trjap	U	I	445	YUNLU 7	Trjap	U	I
336	IR71525-19-1-1	Trjap	U	I	446	IR47686-09-01-B-1	Trjap	U	I
340	IRAT 109	Trjap	U	I	447	IR53236-275-1	Trjap	U	I
341	IRAT 112	Trjap	U	I	449	IR65261-19-1-B	Trjap	U	I
343	IRAT 144	Trjap	U	I	452	IR65907-206-4-B	Trjap	U	I
344	IRAT 170	Trjap	U	I	454	IR71524-44-1-1	Trjap	U	I
345	IRAT 177	Trjap	U	I	457	VIETNAM3	Trjap	U	T
348	IRAT 216	Trjap	U	I	458	IR47684-05-1-B	Trjap	U	I
349	IRAT 234	Trjap	U	I	459	PRIMAVERA	Trjap	U	I
350	IRAT 257	Trjap	U	I	749	CHUAN 3	Trjap	U	No Inf
352	IRAT 362	Trjap	U	I	999	EARLY MUTANT IAC 165	Trjap	U	I

Table S2: correlation matrix between morphological measurements, Ecomeristem model parameters and nonstructural carbohydrates in source leaves estimated for 123 genotypes of the japonica rice diversity panel. Acronyms are described in Table 2.

Variables	NBT	LLL	NBL	DR	SDW	RGR	EPSIB	MGR	PLASTO	SLAP	ICT	DEV_PLASTO_MGR	GLU	FRU	SUC	STA	NSC
NBT	**1**																
LLL	-0.076	**1**															
NBL	**0.817**	-0.079	**1**														
DR	**0.539**	-0.217	**0.667**	**1**													
SDW	**0.499**	**0.463**	**0.438**	**0.287**	**1**												
RGR	**0.588**	**0.266**	**0.628**	**0.653**	**0.584**	**1**											
EPSIB	0.081	0.01	0.037	**0.239**	0.192	0.183	**1**										
MGR	-0.089	**0.731**	-0.077	-0.152	**0.339**	**0.355**	-0.089	**1**									
PLASTO	**-0.423**	0.039	**-0.515**	**-0.724**	**-0.29**	**-0.607**	**-0.233**	**0.319**	**1**								
SLAP	-0.024	-0.185	-0.181	-0.088	0.126	-0.193	0.031	-0.078	0.204	**1**							
ICT	**-0.44**	0.047	**-0.369**	-0.134	**-0.38**	**-0.242**	**0.461**	-0.145	-0.045	**-0.312**	**1**						
DEV_PLASTO_MGR	**-0.417**	**-0.232**	**-0.518**	**-0.709**	**-0.437**	**-0.778**	-0.215	-0.043	**0.933**	**0.248**	0.006	**1**					
GLU	-0.175	0.229	-0.075	-0.039	0.085	0.004	-0.079	0.109	-0.034	-0.01	0.004	-0.076	**1**				
FRU	-0.136	**0.264**	-0.051	-0.053	0.133	0.045	-0.083	0.12	-0.059	0.023	-0.063	-0.109	**0.88**	**1**			
SUC	0.111	0.116	0.092	**0.245**	**0.242**	0.22	-0.04	0.159	-0.112	0.007	-0.153	-0.179	**-0.258**	-0.139	**1**		
STA	-0.128	0.048	-0.14	-0.096	0.025	0.059	-0.044	0.171	0.105	0.056	-0.051	0.042	0.176	0.192	**0.314**	**1**	
NSC	-0.053	0.197	-0.035	0.097	0.213	0.183	-0.08	**0.235**	-0.039	0.033	-0.134	-0.133	**0.296**	**0.37**	**0.734**	**0.777**	**1**

Table S3: Suggestive QTL detected at p-values <5.10^{-4}.

Qtl name	Chr	SNP position	LD region	Trait	MAF	F	p-value	markerR2	# of reported genes in 20kb
**1**	1	792359	792359-2813171	DEV_PLASTO_MGR	0.28	15.59	1.36E-04	0.11	9
**2**	1	859635	859635-937008	NBT	0.11	13.33	4.00E-04	0.10	7
**3**	1	986764		GLU	0.17	13.60	3.47E-04	0.09	8
**4**	1	2280593	2280593-25077287	ICT	0.31	15.17	1.66E-04	0.11	4
**5**	1	2456648		SDW	0.26	14.42	2.38E-04	0.11	4
**6**	1	2803218		PLASTO	0.2	14.03	2.83E-04	0.09	9
**7**	1	2813225		DEV_PLASTO_MGR and PLASTO	0.28	16.03	1.11E-04	0.11	1
**8**	1	2854835		DEV_PLASTO_MGR	0.22	15.75	1.26E-04	0.11	5
**9**	1	2886293		DEV_PLASTO_MGR and PLASTO	0.2	16.11	1.07E-04	0.11	7
**10**	1	6360493	6360493-6360496	FRU	0.46	13.73	3.25E-04	0.09	7
**11**	1	23882306	23882306-23922001	PLASTO	0.38	13.27	4.05E-04	0.09	8
**12**	1	28818609	28818609-28980468	RGR	0.11	14.75	2.01E-04	0.11	3
**13**	1	28985793		RGR	0.14	13.51	3.61E-04	0.10	5
**14**	1	32139722	32139722-32133860	NBT	0.11	13.23	4.19E-04	0.10	6
**15**	1	33336224		FRU	0.4	15.74	1.27E-04	0.11	6
**16**	1	34167105		LLL	0.15	13.03	4.54E-04	0.09	8
**17**	1	42138453	42138453-42169818	RGR	0.13	12.88	4.89E-04	0.10	4
**18**	2	18637296		SDW	0.27	13.11	4.43E-04	0.10	2
**19**	2	18704391		RGR	0.29	16.06	1.09E-04	0.11	4
**20**	2	24494317		MGR	0.25	14.78	1.98E-04	0.10	3
**21**	2	25352210		MGR	0.31	15.80	1.24E-04	0.11	4
**22**	2	32101207		RGR	0.07	15.57	1.37E-04	0.12	8
**23**	2	33806279		NBL	0.27	13.11	4.38E-04	0.10	9
**24**	3	19005645		RGR	0.06	13.26	4.08E-04	0.10	1
**25**	3	32655537	32655537-32969116	MGR	0.07	12.87	4.92E-04	0.09	5
**26**	4	11887993		NBL	0.18	13.61	3.46E-04	0.10	3
**27**	4	14511266		NBT	0.07	13.41	3.85E-04	0.10	3
**28**	4	28119554		SDW	0.3	13.77	3.24E-04	0.10	7
**29**	4	28121876		SDW	0.29	16.05	1.12E-04	0.12	1
**30**	5	1445349		NSC	0.13	13.34	3.93E-04	0.09	4
**31**	5	4080087		SDW	0.33	14.18	2.66E-04	0.10	5
**32**	5	5548160		NBL	0.16	13.04	4.53E-04	0.10	2
**33**	5	13713053	13713053-13748504	NBT	0.07	15.10	1.74E-04	0.11	4
**34**	6	9661428		NBL	0.37	13.89	3.02E-04	0.10	3
**35**	6	9924240		NBL RGR	0.36	14.26	2.54E-04	0.10	5
**36**	6	21231994		SDW	0.11	14.77	2.01E-04	0.11	5
**37**	6	21338769		FRU	0.06	14.06	2.79E-04	0.09	3

Qtl name	Chr	SNP position	LD region	Trait	MAF	F	p-value	markerR2	# of reported genes in 20kb
**38**	7	1500590		MGR	0.12	14.26	2.54E-04	0.10	4
**39**	7	4334575		SLAP	0.17	13.02	4.57E-04	0.10	5
**40**	7	4746733		EPSIB	0.37	14.01	2.86E-04	0.11	8
**41**	7	18923835	18923835-19039440	DR	0.28	13.65	3.39E-04	0.09	6
**42**	7	19160805		DR	0.28	14.79	1.97E-04	0.10	1
**43**	7	19386273	19386273-19397751	DR	0.15	13.31	3.98E-04	0.09	5
**44**	7	19475740		NBL	0.15	14.87	1.90E-04	0.11	3
**45**	7	21628202		FRU	0.33	13.01	4.61E-04	0.09	6
**46**	7	22559297		NSC and PLASTO	0.22	13.10	4.40E-04	0.08	4
**47**	8	18679124		NBL	0.07	13.23	4.14E-04	0.10	3
**48**	9	678173		LLL	0.08	13.24	4.12E-04	0.09	3
**49**	9	10393681		DR	0.11	14.86	1.91E-04	0.10	5
**50**	9	14406563	14406563-14456284	LLL	0.09	16.11	1.07E-04	0.10	6
**51**	9	21275388		DEV_PLASTO_MGR	0.11	12.80	5.08E-04	0.09	6
**52**	10	11542583		EPSIB	0.11	14.87	1.90E-04	0.11	5
**53**	10	15253191		GLU	0.07	14.10	2.74E-04	0.10	12
**54**	10	18059128	18059128-18061638	ICT	0.33	13.63	3.42E-04	0.10	2
**55**	10	21779320		GLU	0.2	13.16	4.28E-04	0.10	5
**56**	11	3161547		NBT	0.32	13.83	3.16E-04	0.10	6
**57**	11	6671341		ICT	0.21	14.78	1.99E-04	0.11	4
**58**	11	7693392	7693392-7693395	GLU	0.09	13.05	4.52E-04	0.09	5
**59**	11	22526446		SDW	0.24	13.86	3.11E-04	0.10	5
**60**	11	23866023		EPSIB	0.42	14.49	2.28E-04	0.11	3
**61**	11	26441049		EPSIB	0.09	13.42	3.78E-04	0.10	5
**62**	11	27158519	27158519-27158522	EPSIB	0.41	13.91	2.99E-04	0.11	6
**63**	12	3643368		PLASTO	0.08	13.17	4.26E-04	0.10	7
**64**	12	12393537		FRU	0.06	13.98	2.89E-04	0.10	2

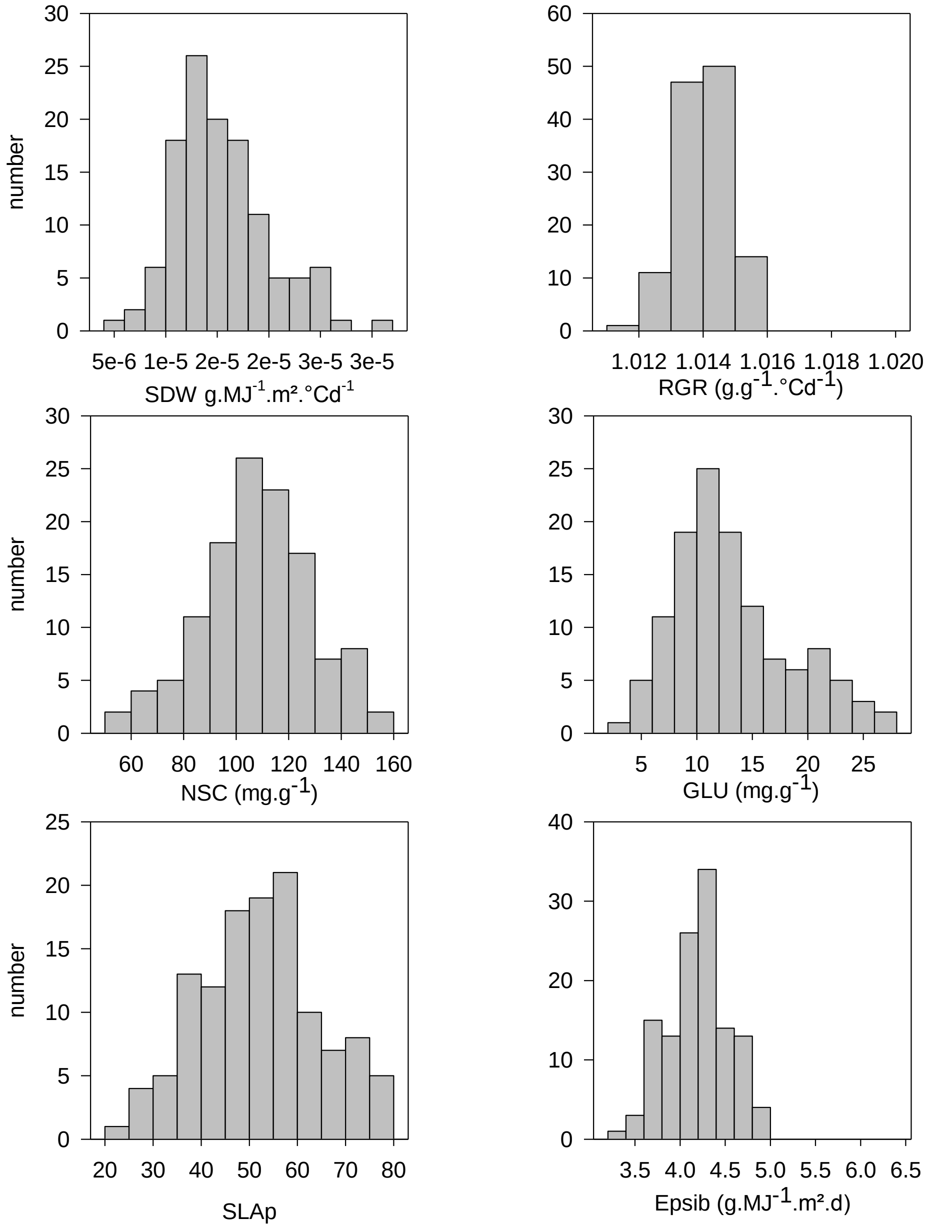

Fig. S1 : Histogram of distribution of other morphological variables and model parameters used for GWAS. Acronyms are explained in Table 2 of the manuscript.

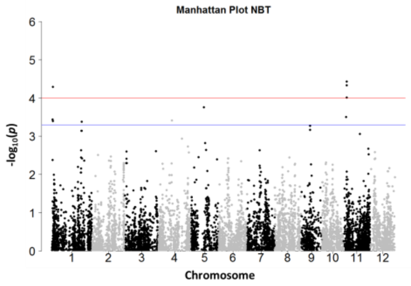
Manhattan Plot NBT
-log10(p)
Chromosome

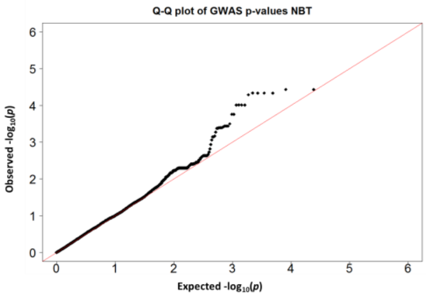
Q-Q plot of GWAS p-values NBT
Observed -log10(p)
Expected -log10(p)

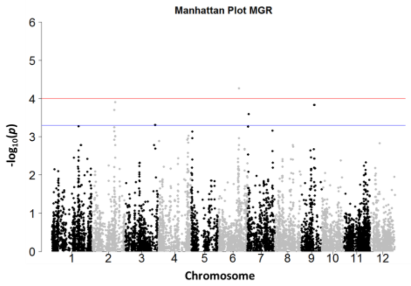
Manhattan Plot MGR
-log10(p)
Chromosome

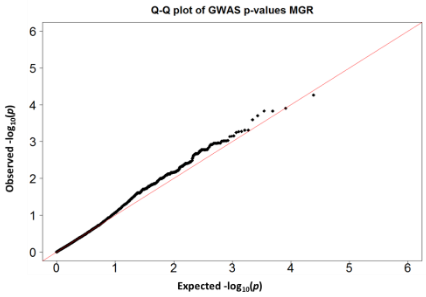
Q-Q plot of GWAS p-values MGR
Observed -log10(p)
Expected -log10(p)

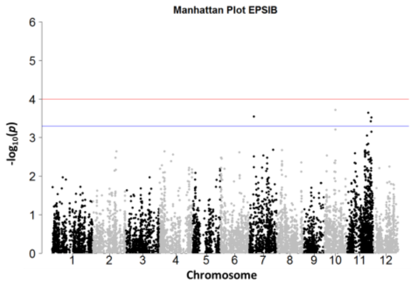
Manhattan Plot EPSIB
-log10(p)
Chromosome

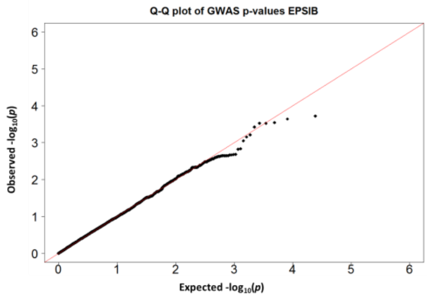
Q-Q plot of GWAS p-values EPSIB
Observed -log10(p)
Expected -log10(p)

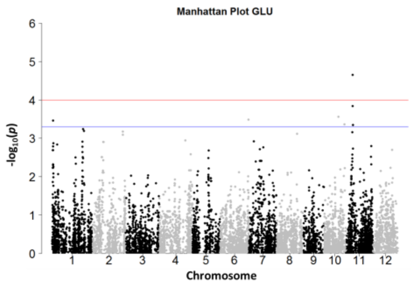
Manhattan Plot GLU
-log10(p)
Chromosome

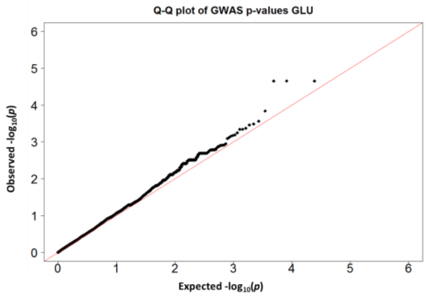
Q-Q plot of GWAS p-values GLU
Observed -log10(p)
Expected -log10(p)

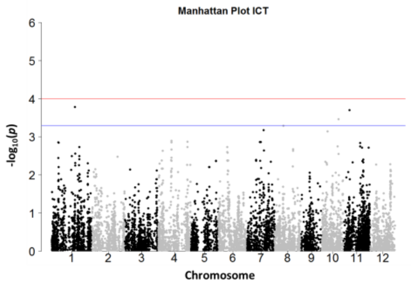
Manhattan Plot ICT
-log10(p)
Chromosome

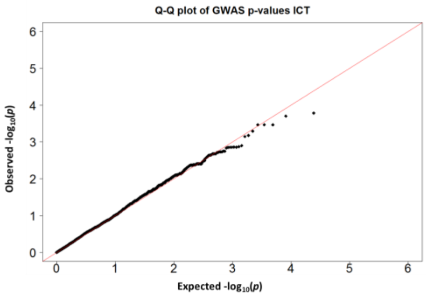
Q-Q plot of GWAS p-values ICT
Observed -log10(p)
Expected -log10(p)

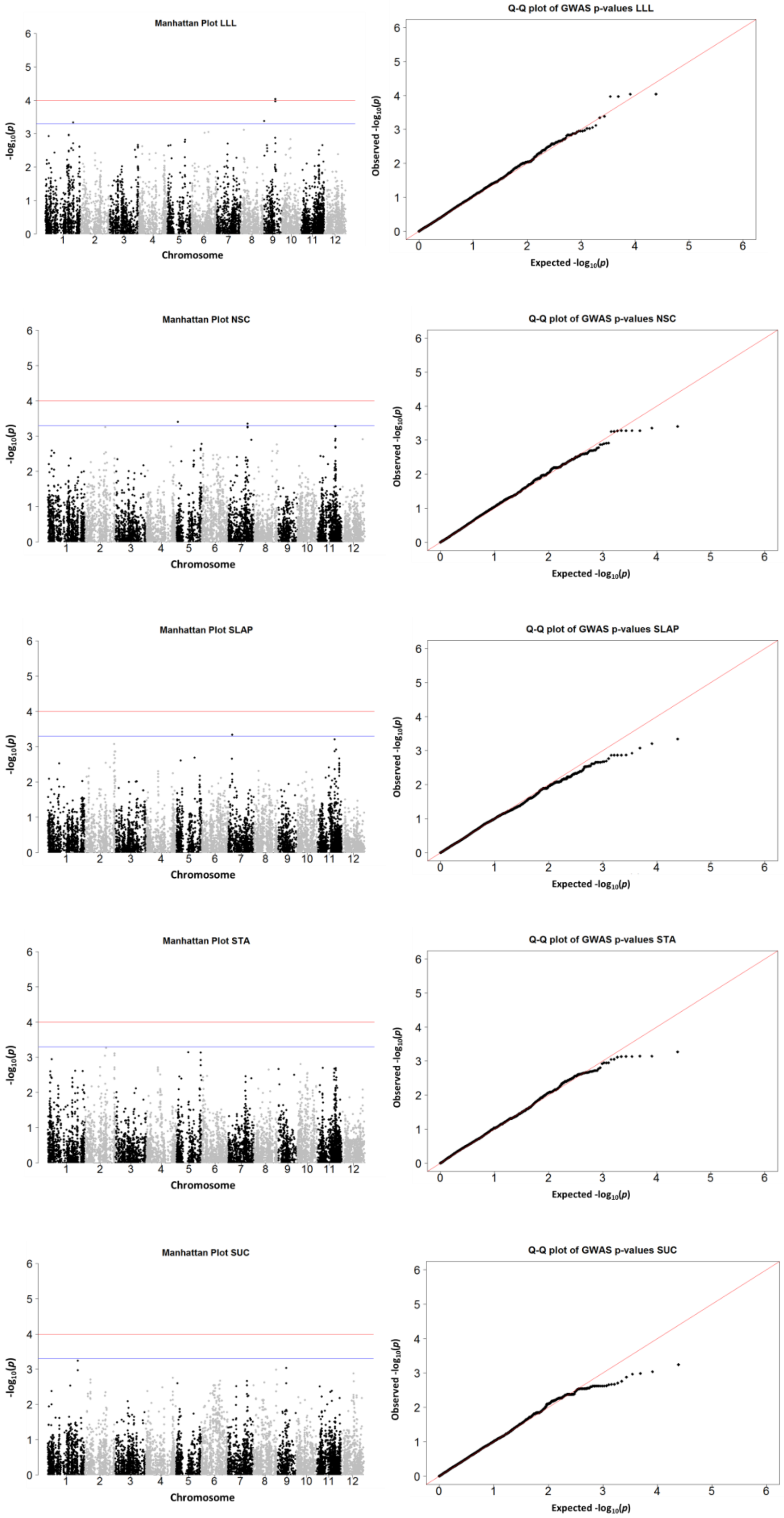

Fig.S2 : Manhattan and Quantile – Quantile Plots for SUC, STA, SLAP, NSC, LLL, ICT, GLU, MGR, NBT and EPSIB. In the Manhattan plots the red line indicates the threshold for significant SNP association ($P<1.10^{-4}$) and the blue line indicates the threshold for suggestive SNP association ($P<5.10^{-4}$).

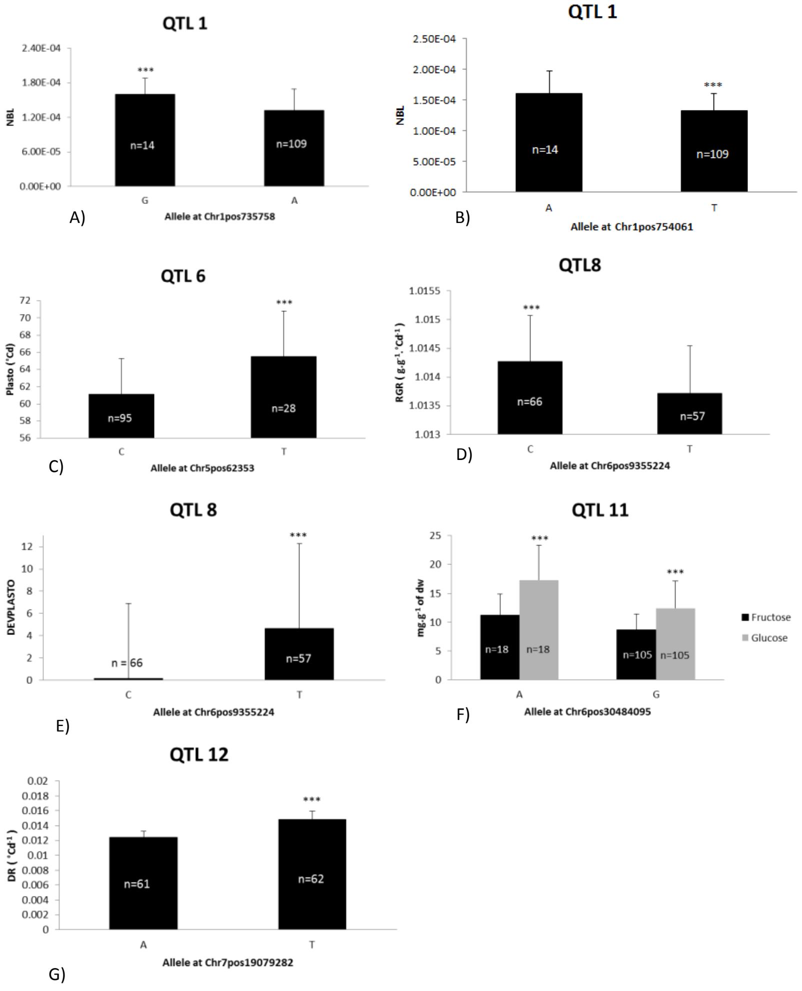

Fig S3. Allele effects at markers associated with non-reported (A-C) and reported (D-G) genes. Phenotypic traits are described in Table 2 and QTL are described in Table 3.
